# Genome-wide identification and analysis of highly specific CRISPR/Cas9 editing sites in pepper (*Capsicum annuum* L.)

**DOI:** 10.1371/journal.pone.0244515

**Published:** 2020-12-29

**Authors:** Guanliang Li, Ziyan Zhou, Lingrui Liang, Zhao Song, Yafei Hu, Junjie Cui, Weili Chen, Kailin Hu, Jiaowen Cheng

**Affiliations:** 1 College of Horticulture, South China Agricultural University, Guangzhou, China; 2 South China Key Laboratory of Urban Agriculture, Ministry of Agriculture and Rural Affairs, Guangzhou, China; 3 Key Laboratory of Biology and Genetic Improvement of Horticultural Crops (South China), Ministry of Agriculture and Rural Affairs, Guangzhou, China; 4 Department of Horticulture, College of Food Science and Engineering, Foshan University, Foshan, Guangdong, China; West China Hospital, Sichuan University, CHINA

## Abstract

The CRISPR/Cas9 system is an efficient genome editing tool that possesses the outstanding advantages of simplicity and high efficiency. Genome-wide identification and specificity analysis of editing sites is an effective approach for mitigating the risk of off-target effects of CRISPR/Cas9 and has been applied in several plant species but has not yet been reported in pepper. In present study, we first identified genome-wide CRISPR/Cas9 editing sites based on the ‘Zunla-1’ reference genome and then evaluated the specificity of CRISPR/Cas9 editing sites through whole-genome alignment. Results showed that a total of 603,202,314 CRISPR/Cas9 editing sites, including 229,909,837 (~38.11%) NGG-PAM sites and 373,292,477 (~61.89%) NAG-PAM sites, were detectable in the pepper genome, and the systematic characterization of their composition and distribution was performed. Furthermore, 29,623,855 highly specific NGG-PAM sites were identified through whole-genome alignment analysis. There were 26,699,38 (~90.13%) highly specific NGG-PAM sites located in intergenic regions, which was 9.13 times of the number in genic regions, but the average density in genic regions was higher than that in intergenic regions. More importantly, 34,251 (~96.93%) out of 35,336 annotated genes exhibited at least one highly specific NGG-PAM site in their exons, and 90.50% of the annotated genes exhibited at least 4 highly specific NGG- PAM sites, indicating that the set of highly specific CRISPR/Cas9 editing sites identified in this study was widely applicable and conducive to the minimization of the off-target effects of CRISPR/Cas9 in pepper.

## Introduction

In mutants, which are of great significance for both gene function analysis and crop genetic improvement, allelic variation mainly results from naturally or artificially induced mutation. Compared to natural variation, the most prominent advantage of artificially induced mutation is the high mutation frequency achieved. The main methods currently used for achieving artificially induced mutation include physical mutagenesis, chemical mutagenesis, random transposon insertion, and target gene editing technologies. Among these approaches, target gene editing, in which nucleotide variation is introduced at an appointed site and the target mutations are obtained accurately and efficiently, thereby speeding up the functional identification of target genes and genetic breeding improvement, is an ideal method for artificially inducing mutations [1].

A variety of target gene editing techniques, including the use of zinc-finger nucleases (ZFNs), transcription activator-like effector nucleases (TALENs) and the CRISPR/Cas system, have been developed to date [2]. The CRISPR/Cas system, which has entered the mainstream in recent years and been widely used in humans [3], animals [4], microorganisms [5] and plants [6], possesses the outstanding advantages of high simplicity and efficiency in contrast to the other two techniques. According to the number and functional characteristics of the Cas gene, CRISPR/Cas systems can be divided into 2 categories, including 6 different types (I to VI) [7–9]. The first category of CRISPR/Cas systems, including types I, III and IV, requires multiple Cas proteins to collaboratively interfere with the target gene, while the second category requires only one Cas protein. The type II CRISPR/Cas system, namely CRISPR/Cas9 system belongs to the second category and is now the most widely used gene editing system.

The CRISPR/Cas9 gene editing system is mainly composed of one Cas9 protein and one small guide RNA (sgRNA). The Cas9 protein from *Streptococcus pyogenes* (SpCas9) was first applied for use in the CRISPR/Cas9 system [10]; SpCas9 recognizes the protospacer adjacent motif (PAM) sequence 5′-NGG-3′ (where “N” can be any nucleotide base) in the target DNA, then cleaves the target DNA at 3 nt upstream of the PAM site, generating a blunt end, and gene editing is finally achieved by nucleotide insertion, deletion and substitution at the cleavage site mediated by the receptor cellular DNA repair machinery, including the nonhomologous end joining (NHEJ) and homologous recombination repair (HDR) mechanisms [11]. The sgRNA of the CRISPR/Cas9 system, artificially designed based on crRNA (CRISPR RNA) and the core sequence of trans-acting crRNA (tracrRNA), is a short single-stranded RNA that guides the Cas9/sgRNA complex to perform cleavage at 3 nt upstream of the PAM site through complementary base pairing between the 5’ end (~20 bp) of the sgRNA and the protospacer sequence of the target DNA, which determines the specificity of gene editing [12].

Previous studies have found that even if the sgRNA imperfectly matches the protospacer, the Cas9 protein can still perform cleavage at 3 nt upstream of the PAM site, making gene editing possible in nontarget regions; thus, off-target effects can occur [13–15]. To reduce or eliminate the risk of off-target effect, the identification of candidate editing sites with high specificity is a prerequisite for the application of the CRISPR/Cas9 system. To date, a variety of tools based on whole-genome sequence similarity analysis have been developed for target site design and off-target risk assessment, such as CrisprGE [16], Cas-OFFinder [17], Cas-Designer [18], CRISPRdirect [19] and CRISPOR [20]. However, the majority of those tools have been mainly applied in humans and animals. Based on whole-genome reference sequences, the distribution and specificity of genome-wide CRISPR/Cas9 editing sites in *Arabidopsis thaliana*, *Medicago truncatula*, soybean (*Glycine max*), tomato (*Solanum lycopersicum*), *Brachypodium distachyon*, rice (*Oryza sativa*), *Sorghum bicolor*, maize (*Zea mays*) and grape (*Vitis vinifera*) have been systematically analysed and compared [12, 21], providing an important reference for choosing highly specific editing sites of related species.

Pepper (*Capsicum* spp.) belongs to the family *Solanaceae* and has a cosmopolitan distribution and considerable economic importance [22]. The reference genome sequences of pepper were first released in 2014 [23, 24], marking the transition of pepper research from structural genomics to functional genomics. The identification and functional analysis of important genes controlling agronomic traits have become a significant direction in molecular genetics research in pepper. With the development and continuous improvement of technologies for pepper regeneration *in vitro* and its genetic transformation [25, 26], the CRISPR/Cas9 gene editing system will become a powerful tool and will be widely used for the functional analysis of pepper genes. In this study, we first identified CRISPR/Cas9 editing sites at the genome-wide level in pepper and then evaluated the obtained specificity through whole-genome sequence alignment. The purpose of this study was to provide a reference for the selection of highly specific CRISPR/Cas9 editing sites and facilitate the application of CRISPR/Cas9-mediated gene editing in pepper.

## Materials and methods

### Genomic data and CRISPR/Cas9 editing site identification

The ‘Zunla-1’ (v2.0) pepper reference genome sequence and related genome annotations [23] were used for CRISPR/Cas9 editing site identification. There were two PAM sites recognized by the CRISPR/Cas9 system: 5'-NGG-3' and 5'-NAG-3', which were identified by using EMBOSS software [27] in both the positive and reverse strands of the Zunla-1 reference genome sequence. The 20-nt sequences before all 5'-NGG-3' and 5'-NAG-3' sites were extracted to form two protospacer sets, referred to as the GG_spacer set and AG-spacer set, respectively.

### Identification of highly specific CRISPR/Cas9 editing sites

Based on the method reported previously, the specificity of CRISPR/Cas9 editing sites in pepper was evaluated. Class 0.0 and Class 1.0 spacers were expected to provide high specificity in CRISPR/Cas9 gene editing [12] and were thus classified as highly specific sites in this study. Since the sgRNA/Cas9 complex showed much less affinity and tolerance toward mismatches at the NAG-PAM site [5], in this study, we only assessed the specificity of the GG_spacers, for which the possibility of off-target effects was evaluated by using the AG_spacer set. The method is outlined as follows:

1. The hard-masking function of USEARCH [28] was used to mask and remove GG_spacers containing low-complexity sequences;
2. GG_spacers with the same sequences at the 6~20-nt region were removed;
3. GASSST [29] and UBLAST [28] were used to generate a pairwise alignment for the remaining GG_spacers. According to the GG_spacer position and the minimum number of mismatches (minMM_GG, including InDel and SNP) between each GG_spacer and other GG_spacers, the GG_spacers were graded into three classes: Class 0 spacers shared no significant matching sequence with other GG_spacers; Class 1 spacers showed no fewer than four mismatches (minMM_GG≥4) or three mismatches adjacent to PAM sites; Class 2 included the other GG_spacers;
4. For Class 0 and Class 1 GG_spacers, pairwise alignments were performed with AG_spacers, which were further graded into four classes as follows according to their position and the minimum number of mismatches (minMM_GG, including InDel and SNP) between each GG_spacer and other AG-spacers: Class 0.0 spacers exhibited no fewer than three mismatches with AG_spacers (minMM_AG≥3) or shared no significant matching sequence with AG_spacers; Class 0.1 spacers exhibited fewer than three mismatches with AG_spacers; Class 1.0 spacers exhibited no fewer than three mismatches with AG_spacers (minMM_AG≥3) or shared no significant matching sequence with AG_spacers; Class 1.1 spacers exhibited fewer than three mismatches with AG_spacers.

### PCR verification and sequence analysis

Primer pairs flanking the selected target sites were designed by using the Primer3web (version 4.1.0; http://primer3.ut.ee/) tool. PCR reaction was performed in a 20 μL mixture including 2.0 μL DNA template (50 ng/μL), 2.0 μL PCR buffer (10×), 2.0 μL Mg2+ (25 mM), 1.5 μL forward and reverse primer (1 μM), 0.2 μL dNTPs (10 mM), and 1U Taq DNA polymerase. PCR procedure was as follow: 94°C for 3 min, 32 cycles of 94°C for 30 s, 55°C for 30 s, and 1 min at 72°C; and a final extension at 72°C for 10 min. PCR amplication of each sites were repeated three times and then the products were directly sequenced and assembled. Alignment of each sequence to the reference genome was conducted by using the local blastn:2.9.0+.

## Results and discussion

### Content and composition of CRISPR/Cas9 editing sites in pepper genome

A total of 603,202,314 CRISPR/Cas9 editing sites, containing 229,909,837 (~38.11%) NGG-PAM sites and 373,292,477 (~61.89%) NAG-PAM sites, were detected in the pepper genome. This was approximately 4.63 times greater than the number identified in another *Solanaceae* species, tomato (130,302,150), conforming to the law that the larger the size of a genome, the greater the number of CRISPR/Cas9 editing sites it contains [12]. The average density of NGG-PAM and NAG-PAM in pepper was 69.75/Kb and 112.56/Kb (Table 1), respectively, which were similar to those in tomato (63.30/Kb and 103.43/Kb, respectively), but the density of NGG-PAM in pepper was much less than that in monocot species such as rice (101.69/Kb) and maize (119.22/Kb) [12].

**Table 1:** The number and density of NGG-PAM and NAG-PAM sites on pepper chromosomes.

Chr.	NGG		NAG		Subtotal
	No.	Density	No.	Density	
P1	22,489,579	74.71	33,673,230	111.86	56,162,809
P2	11,839,695	72.21	18,417,689	112.33	30,257,384
P3	17,618,433	67.37	29,560,783	113.04	47,179,216
P4	15,393,261	71.36	24,658,517	114.32	40,051,778
P5	15,303,392	70.43	24,853,200	114.39	40,156,592
P6	15,305,364	69.72	25,109,431	114.38	40,414,795
P7	15,309,150	68.93	24,810,273	111.70	40,119,423
P8	11,278,024	73.57	17,545,055	114.45	28,823,079
P9	16,539,878	69.26	27,445,080	114.93	43,984,958
P10	14,506,128	70.51	23,477,296	114.11	37,983,424
P11	15,159,289	68.80	24,592,215	111.61	39,751,504
P12	15,974,353	69.47	26,241,216	114.12	42,215,569
P0	43,193,291	60.43	72,908,492	102.00	116,101,783
Total	229,909,837	69.75	373,292,477	112.56	603,202,314

**Fig 1:**
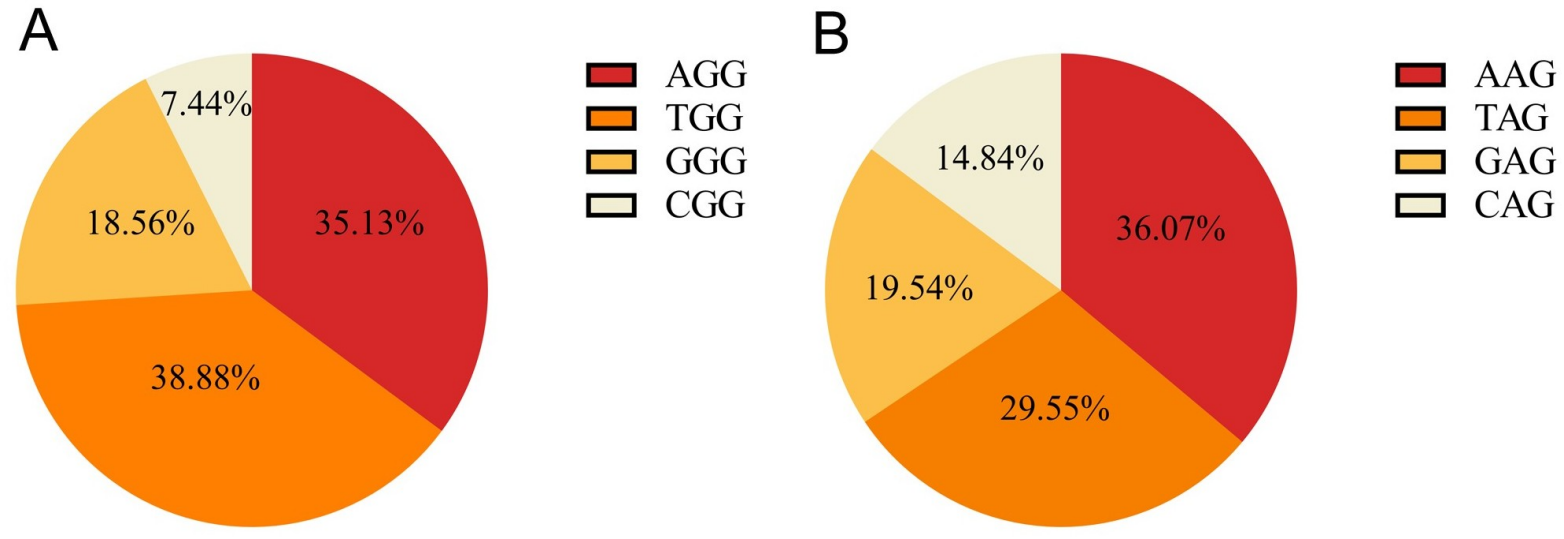
Composition of pepper PAM sites. A, NGG-PAM; B, NAG-PAM.

With respect to the composition of the PAM sites, the TGG and CGG types accounted for the highest (~38.88%) and lowest proportions (~7.44%) of total NGG-PAM sites, respectively (Fig 1A), similar to the composition pattern found in the grape genome [21]. For NAG-PAM sites, the AAG type was the most abundant, with a proportion of ~36.07%, followed by TAG, GAG and CAG, accounting for 29.55%, 19.54% and 14.84% of the total NAG-PAM sites, respectively (Fig 1B).

### Distribution characteristics of CRISPR/Cas9 editing sites in pepper genome

**Fig 2:**
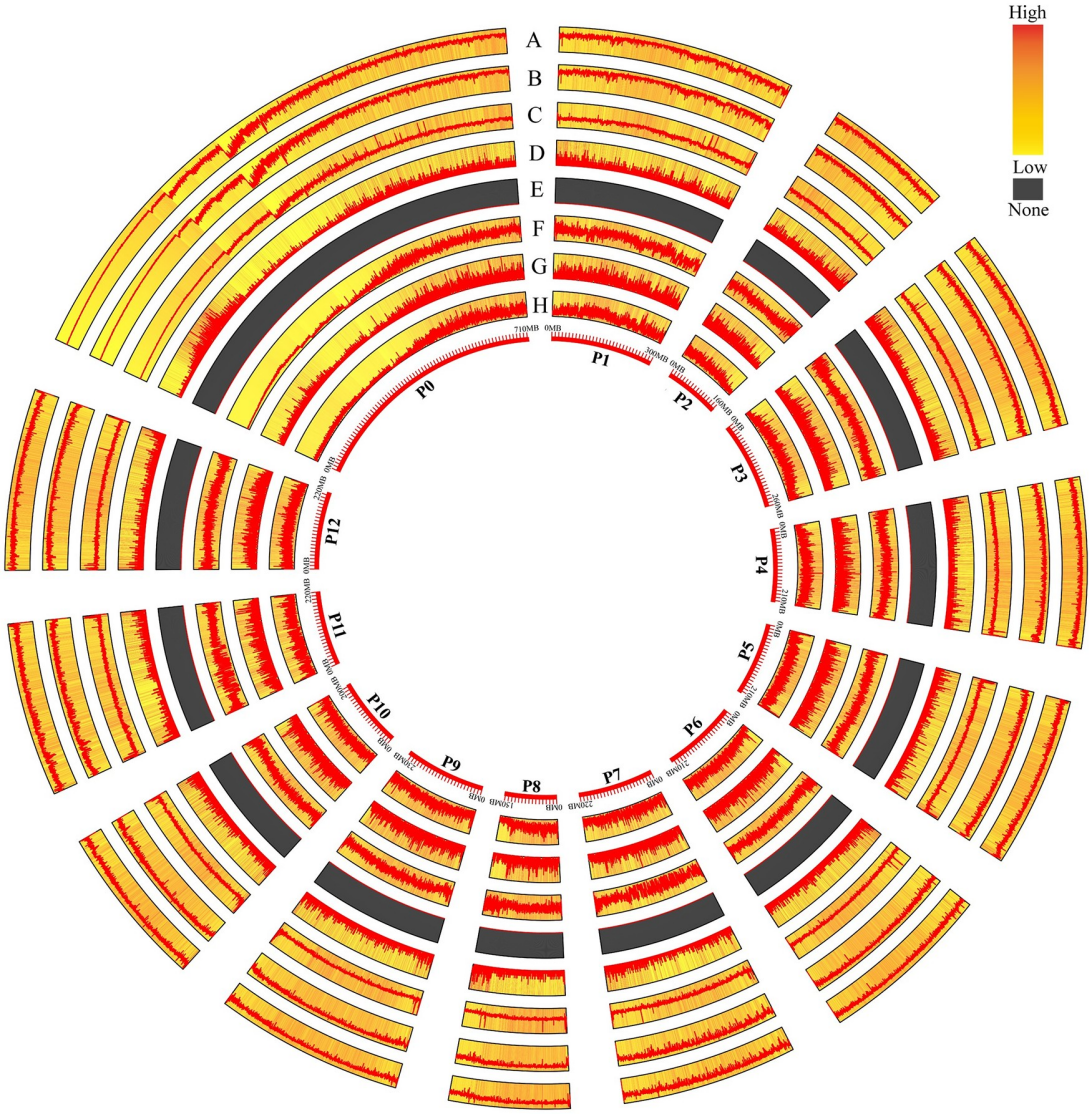
Distribution of different kinds of CRISPR/Cas9 editing sites in the pepper genome. A, NGG-PAM+NAG-PAM site; B, NGG-PAM site; C, NAG-PAM site; D, Class 0.0; E, Class 0.1; F, Class 1.0; G, Class 1.1; H, Class 2.

**Fig 3:**
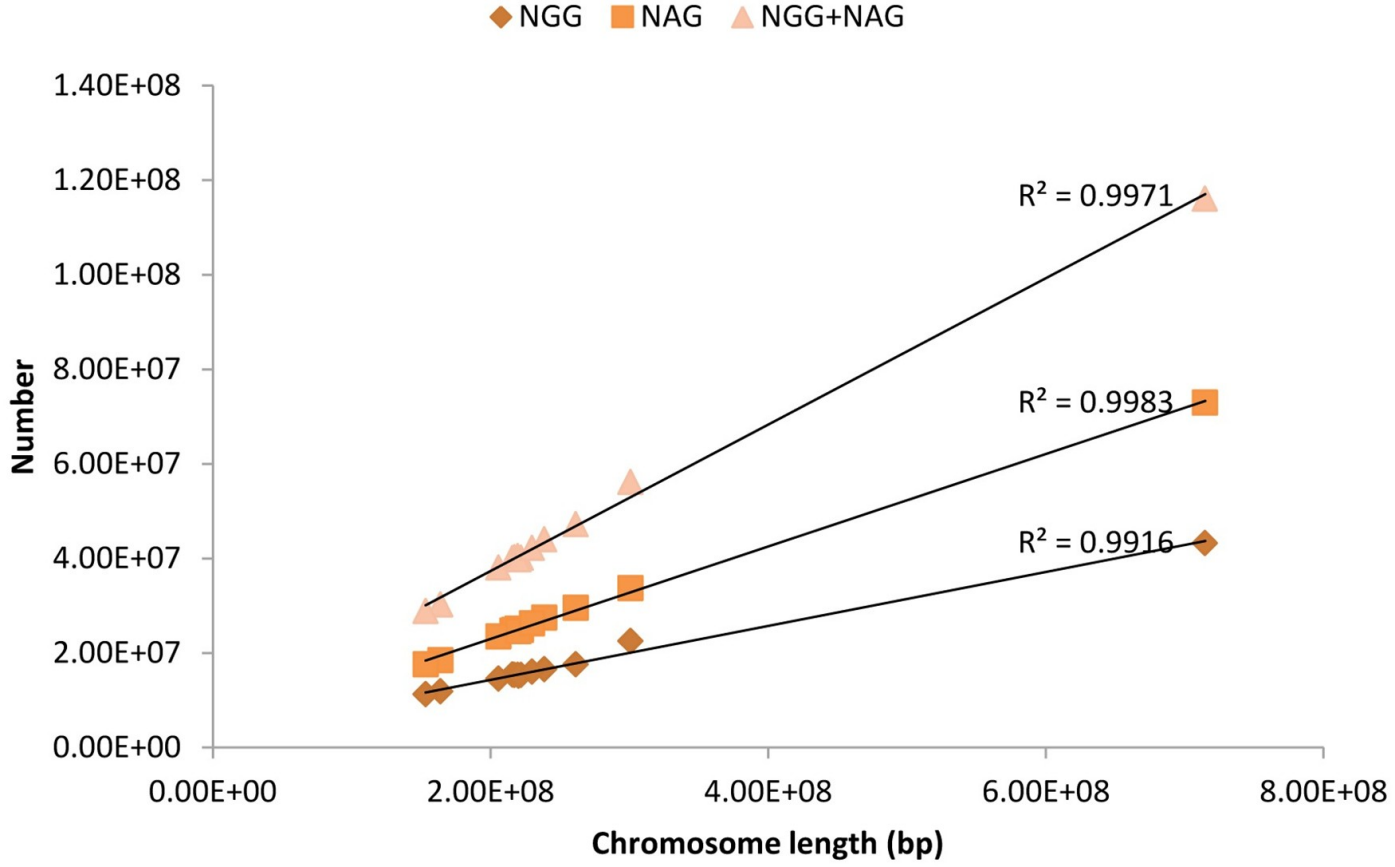
Correlation between the number of CRISPR/Cas9 editing sites and chromosome length in pepper.

The CRISPR/Cas9 editing sites (NGG-PAM and NAG-PAM) were uniformly distributed on all 12 chromosomes (P1~P12) of pepper (Fig 2). With the exception of chromosome P0, P1 and P8 exhibited the most and least CRISPR/Cas9 editing sites, respectively (Table 1). The number of NGG-PAM and NAG-PAM sites on the pepper chromosomes was significantly positively correlated (R^2^ = 0.997) with chromosome length (Fig 3). The density of NGG-PAM sites on different chromosomes (not including P0) ranged from 67.37/Kb (chromosome P3) to 74.71/Kb (chromosome P1). The densities of NAG-PAM sites on different chromosomes (excluding P0) were relatively similar to each other, with the minimum and maximum densities of 111.61/Kb (P11) and 114.93/Kb (P9), respectively (Table 1).

**Fig 4:**
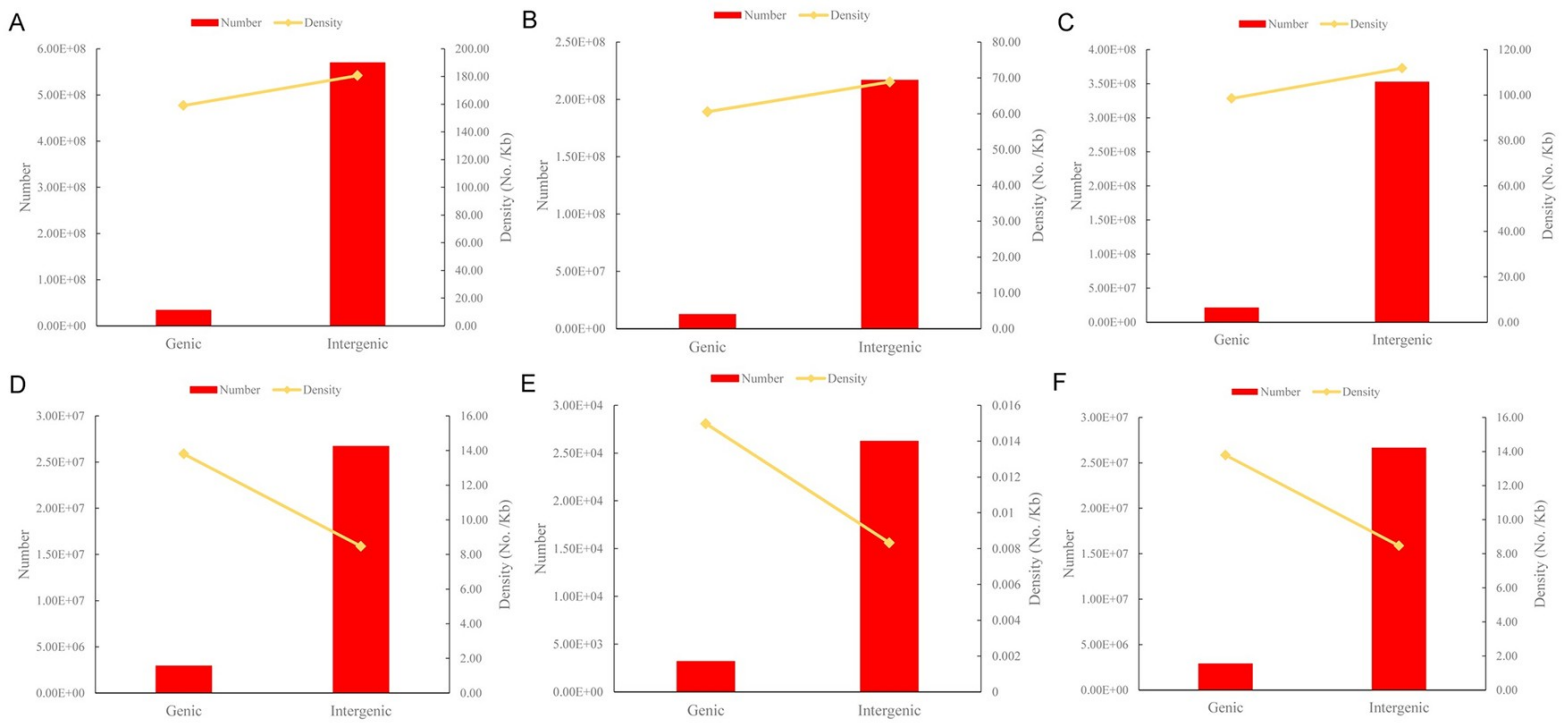
Comparison of the number and density of CRISPR/Cas9 editing sites between genic and intergenic regions. A, NGG-PAM+NAG-PAM site; B, NGG-PAM site; C, NAG-PAM site; D, Class 0.0+Class 1.0; E, Class 0.0; F, Class 1.0.

The vast majority of NGG-PAM (~94.41%) and NAG-PAM (~94.42%) sites were located in the intergenic regions of the pepper genome, while 8,661,656 (~3.77%) and 3,425,476 (~1.49%) NGG-PAM sites were located in intron and exon regions, respectively, and the rest (~0.32%) were located in UTRs and splicing regions (Table 2). Regarding the distribution pattern in different genomic regions, the pattern of NAG-PAM sites was similar to that of NGG-PAM sites (Table 2). The density of CRISPR/Cas9 editing sites in genic regions (including UTRs, exons, introns and splicing sites,) was lower than that in intergenic regions for NGG+NAG-PAM (159.03/Kb versus 180.68/Kb, Fig 4A), NGG-PAM (60.55/Kb versus 68.87/Kb, Fig 4B) and NAG-PAM (98.49/Kb versus 111.81/Kb, Fig 4C), which differs from the situation in grape [21].

**Table 2:** The number of CRISPR/Cas9 editing sites in different genomic regions.

Genomic Region	NGG+NAG		NGG		NAG	
	No.	Percentage	No.	Percentage	No.	Percentage
Intergenic	569,505,881	94.41%	217,081,038	94.42%	352,424,843	94.41%
5'UTR	975,566	0.16%	373,564	0.16%	602,002	0.16%
3'UTR	937,645	0.16%	340,106	0.15%	597,539	0.16%
Exon	8,487,423	1.41%	3,425,476	1.49%	5,061,947	1.36%
Intron	23,217,393	3.85%	8,661,656	3.77%	14,555,737	3.90%
Splicing	78,406	0.01%	27,997	0.01%	50,409	0.01%
Total	603,202,314	100.00%	229,909,837	100.00%	373,292,477	100.00%

### Content of highly specific NGG-PAM sites in pepper genome

Through filtering and alignment analysis, 30,402,397 (~13.22%) NGG-PAM sites were successfully graded based on their specificity (Table 3). The total number of highly specific NGG-PAM sites in pepper, including those belonging to Class 0.0 and Class 1.0, was 29,623,855, which was 4.50 times higher than that in tomato, accounting for ~12.88% of the total NGG-PAM sites (Table 3), which was in line with the general rule that the number of specific gRNA spacers is positively correlated with genome size in eudicot species [12]. On average, there were 8.81/Kb highly specific sites in the pepper genome, which is comparable to that in the tomato genome (8.42/Kb, Table 3).

**Table 3:** The number of NGG-PAM sites with differences in specificity on pepper chromosome.

Chr.	Class 0.0	Class 1.0	Highly specific*		Class 1.1	Class 2	Subtotal
			No.	Density			
P1	2,707	2,782,878	2,785,585	9.25	7,070	64,000	2,856,655
P2	1,795	1,631,097	1,632,892	9.96	3,625	33,562	1,670,079
P3	2,640	2,648,954	2,651,594	10.14	5,968	57,210	2,714,772
P4	1,962	2,193,502	2,195,464	10.18	5,542	51,577	2,252,583
P5	1,957	2,136,291	2,138,248	9.84	5,550	52,706	2,196,504
P6	2,091	2,211,372	2,213,463	10.08	5,509	51,797	2,270,769
P7	1,758	1,862,107	1,863,865	8.39	4,909	44,974	1,913,748
P8	1,443	1,643,765	1,645,208	10.73	3,616	36,134	1,684,958
P9	2,121	2,403,863	2,405,984	10.08	6,308	59,730	2,472,022
P10	1,949	2,041,454	2,043,403	9.93	5,316	49,347	2,098,066
P11	1,788	1,905,155	1,906,943	8.65	4,871	46,188	1,958,002
P12	2,124	2,271,133	2,273,257	9.89	5,621	55,596	2,334,474
P0	5,072	3,862,877	3,867,949	5.41	11,075	100,741	3,979,765
Total	29,407	29,594,448	29,623,855	8.81	74,980	703,562	30,402,397

*, equal to the sum of Class 0.0 and Class 1.0; the number of Class 0.1 spacers on all chromosomes is 0.

**Fig 5:**
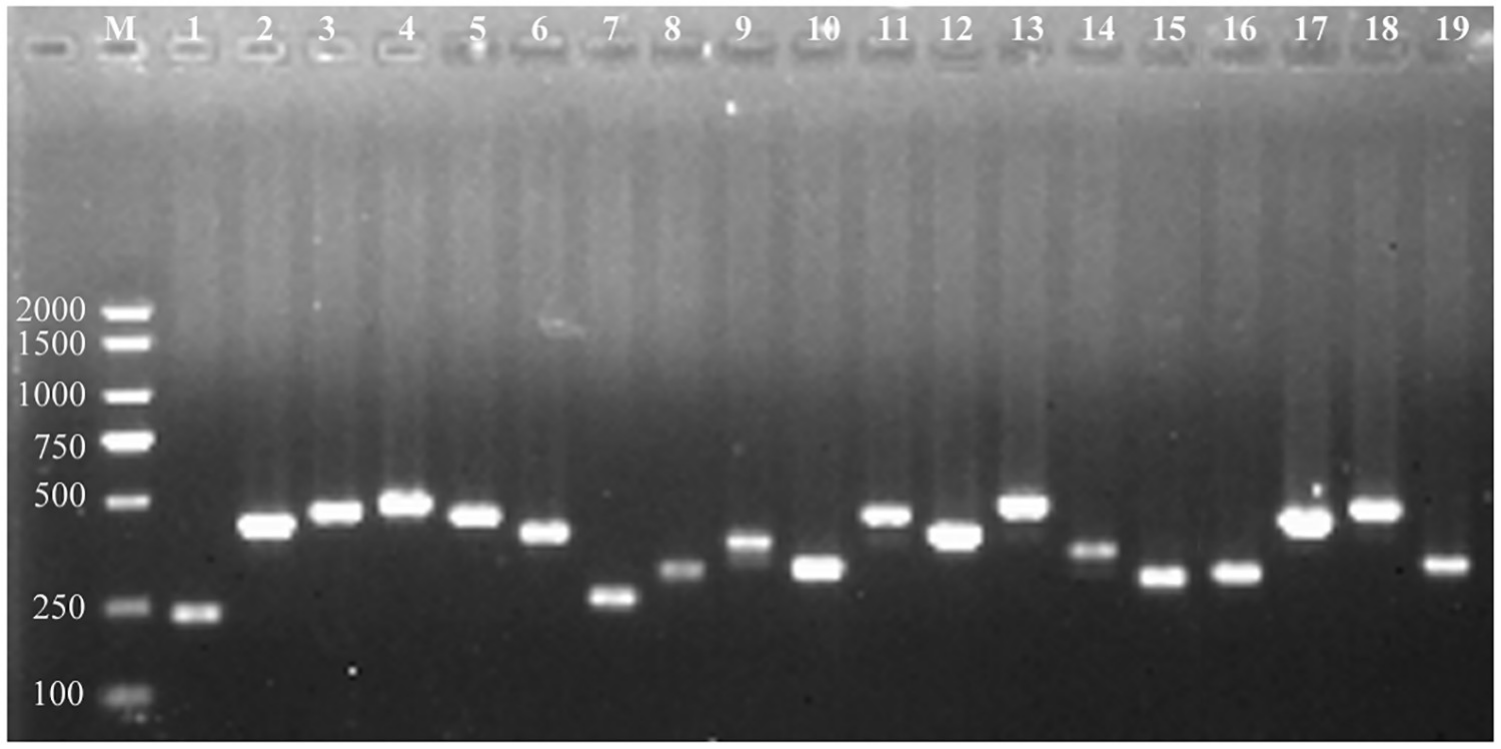
PCR amplification of 19 highly-specific target sites. M, DL2000 plus, 1 to 10 represent A1 to A10 belonging to class0.0; 11 to 19 represent B1 to B9 belonging to class 1.0 (S1 Table).

To validate the specificity of target sites belonging to the class 0.0 and class 1.0, a random set of 19 sites were chosen to be amplified by PCR, and then the PCR products were directly sequenced and assembled. After aligning them back to the Zunla-1 reference genome, all of the products were matched to one unique location in the genome (Fig 5, S1 Table and S1 Fig), indicating that the target sites of class 0.0 and class 1.0 had low risk of off-target.

### Characterization of highly specific NGG-PAM sites’ distribution in pepper genome

The highly specific NGG-PAM sites were evenly distributed on all 12 chromosomes (P1~P12) of pepper (Fig 2). With the exception of P0, chromosomes P1 and P2 contained the maximum and minimum number of highly specific NGG-PAM sites, respectively (Table 3). The number of highly specific NGG-PAM sites in different genomic regions is shown in Table 4. Similar to the distribution of all NGG-PAM sites, there were a total of 26,699,387 (~90.13%) highly specific NGG-PAM sites located in intergenic regions, which was 9.13 times greater than the number in genic regions (Fig 4D). However, the average density of highly specific NGG-PAM sites in genic regions was higher than that in intergenic regions on the whole (13.80/Kb versus 8.47/Kb, Fig 4D) for Class 0.0 (0.015/Kb versus 0.008/Kb, Fig 4E) and Class 1.0 (13.79/Kb versus 8.46/Kb, Fig 4F). The same phenomenon occurs in the grape genome [21].

**Table 4:** The number of highly specific NGG-PAM sites in different genomic regions.

Genomic Region	Class 0.0		Class 1.0		Total	
	No.	Percentage	No.	Percentage	No.	Percentage
Intergenic	26,234	89.21%	26,673,153	90.13%	26,699,387	90.13%
5'UTR	255	0.87%	91,403	0.31%	91,658	0.31%
3'UTR	107	0.36%	99,099	0.33%	99,206	0.33%
Exon	450	1.53%	939,750	3.18%	940,200	3.17%
Intron	2,347	7.98%	1,783,696	6.03%	1,786,043	6.03%
Splicing	14	0.05%	7,347	0.02%	7,361	0.02%
Total	29,407	100.00%	29,594,448	100.00%	29,623,855	100.00%

**Fig 6:**
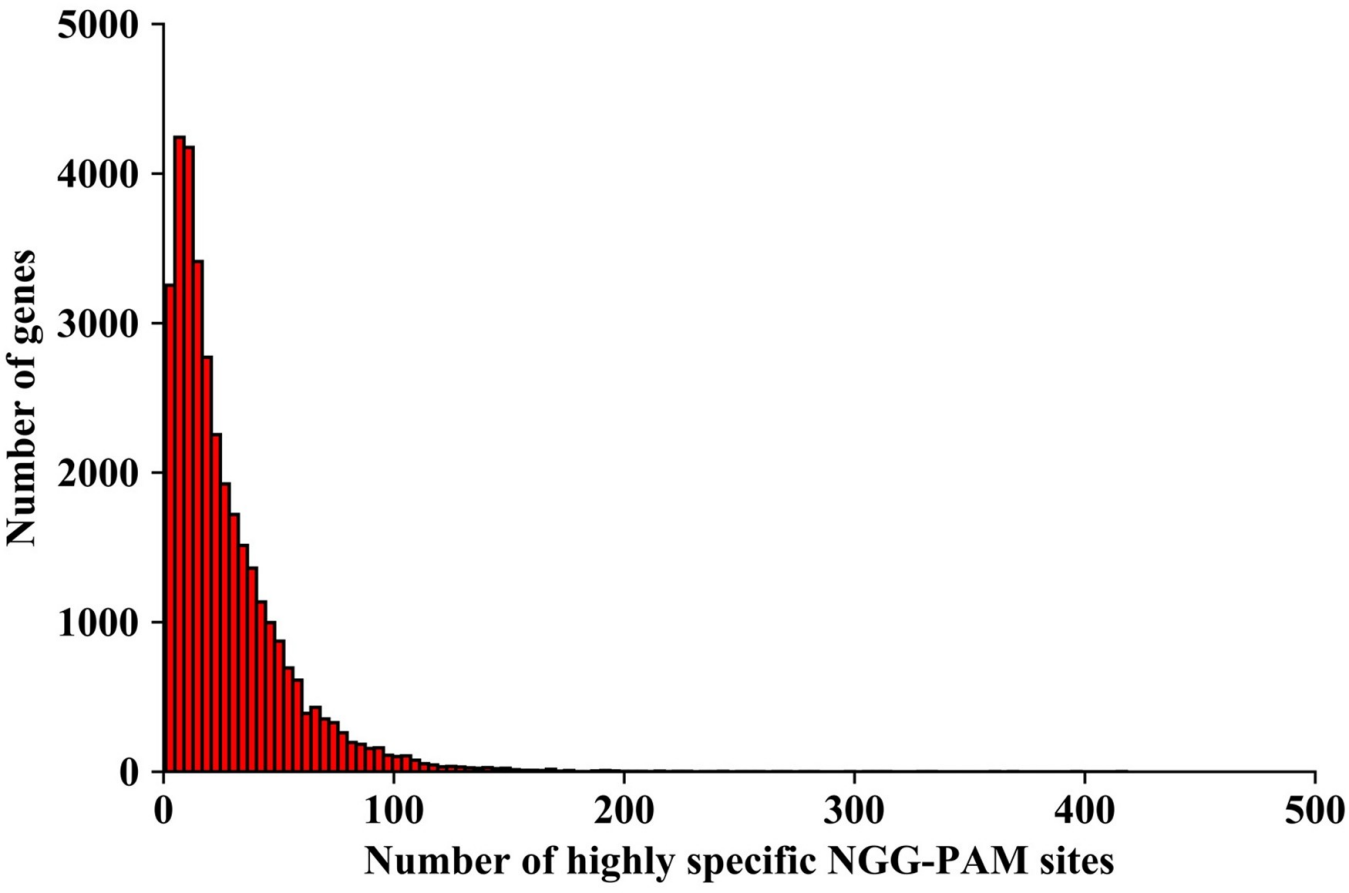
Histogram plots of gene numbers according to the number of exon-targeted highly specific NGG-PAM sites.

We calculated the percentage of annotated genes that contained highly specific NGG-PAM sites identified in this study and found that 34,251 (~96.93%) out of 35,336 annotated genes exhibited at least one highly specific NGG-PAM site in their exons, and 90.50% of annotated genes exhibited at least 4 highly specific NGG- PAM sites (Fig 6 and S2 Table), indicating that the set of highly specific CRISPR/Cas9 editing sites identified in this study was widely applicable and will contribute to the minimization of off-target effects of CRISPR/Cas9 in pepper.

## Supporting information

S1 Table Blast results of a random set of 19 highly-specific editing sites. (XLSX)

S2 Table The number of highly specific NGG-PAM sites in the exons of annotated pepper genes. (XLSX)

S1 Fig Alignment of B7-1 sequence to the Zunla-1 reference genome. (DOCX)

S1 Raw images (PDF)

## References

[1] Liu Y, Li G, Zhang Y, Chen L. Current advances on CRISPR/Cas genome editing technologies in plants. Journal of South China Agricultural University. 2019;40(5):38–49.

[2] Gaj T, Gersbach CA, Barbas CF. ZFN, TALEN, and CRISPR/Cas-based methods for genome engineering. Trends in Biotechnology. 2013;31(7):397–405. 10.1016/j.tibtech.2013.04.004 23664777 PMC3694601

[3] Cho SW, Kim S, Kim JM, Kim JS. Targeted genome engineering in human cells with the Cas9 RNA-guided endonuclease. Nature biotechnology. 2013;31(3):230–2. Epub 2013/01/31. 10.1038/nbt.2507 . 23360966

[4] Hwang WY, Fu Y, Reyon D, Maeder ML, Tsai SQ, Sander JD, et al Efficient genome editing in zebrafish using a CRISPR-Cas system. Nature Biotechnology. 2013;31(3):227–9. 10.1038/nbt.2501 23360964 PMC3686313

[5] Jiang W, Bikard D, Cox D, Zhang F, Marraffini LA. RNA-guided editing of bacterial genomes using CRISPR-Cas systems. Nature biotechnology. 2013;31(3):233–9. 10.1038/nbt.2508 23360965 PMC3748948

[6] Wang M, Mao Y, Lu Y, Wang Z, Tao X, Zhu JK. Multiplex gene editing in rice with simplified CRISPR-Cpf1 and CRISPR-Cas9 systems. J Integr Plant Biol. 2018;60(8):626–31. Epub 2018/05/16. 10.1111/jipb.12667 . 29762900

[7] Shmakov S, Smargon A, Scott D, Cox D, Pyzocha N, Yan W, et al Diversity and evolution of class 2 CRISPR-Cas systems. Nat Rev Microbiol. 2017;15(3):169–82. Epub 2017/01/24. 10.1038/nrmicro.2016.184 . 28111461 PMC5851899

[8] Makarova KS, Koonin EV. Annotation and Classification of CRISPR-Cas Systems. Methods Mol Biol. 2015;1311:47–75. Epub 2015/05/20. 10.1007/978-1-4939-2687-9_4 . 25981466 PMC5901762

[9] Yan WX, Hunnewell P, Alfonse LE, Carte JM, Keston-Smith E, Sothiselvam S, et al Functionally diverse type V CRISPR-Cas systems. Science. 2019;363(6422):88–91. 10.1126/science.aav7271 30523077 PMC11258546

[10] Jinek M, Chylinski K, Fonfara I, Hauer M, Doudna JA, Charpentier E. A Programmable Dual-RNA–Guided DNA Endonuclease in Adaptive Bacterial Immunity. Science. 2012;337(6096):816–21. 10.1126/science.1225829 22745249 PMC6286148

[11] Yao X, Wang X, Hu X, Liu Z, Liu J, Zhou H, et al Homology-mediated end joining-based targeted integration using CRISPR/Cas9. Cell Research. 2017;27(6):801–14. 10.1038/cr.2017.76 28524166 PMC5518881

[12] Xie K, Zhang J, Yang Y. Genome-wide prediction of highly specific guide RNA spacers for CRISPR–Cas9-mediated genome editing in model plants and major crops. Molecular plant. 2014;7(5):923–6. 10.1093/mp/ssu009 24482433

[13] Hsu PD, Scott DA, Weinstein JA, Ran FA, Konermann S, Agarwala V, et al DNA targeting specificity of RNA-guided Cas9 nucleases. Nature biotechnology. 2013;31(9):827–32. 10.1038/nbt.2647 23873081 PMC3969858

[14] Fu Y, Foden JA, Khayter C, Maeder ML, Reyon D, Joung JK, et al High-frequency off-target mutagenesis induced by CRISPR-Cas nucleases in human cells. Nature biotechnology. 2013;31(9):822–6. Epub 2013/06/25. 10.1038/nbt.2623 . 23792628 PMC3773023

[15] Pattanayak V, Lin S, Guilinger JP, Ma E, Doudna JA, Liu DR. High-throughput profiling of off-target DNA cleavage reveals RNA-programmed Cas9 nuclease specificity. Nature biotechnology. 2013;31(9):839–43. Epub 2013/08/13. 10.1038/nbt.2673 . 23934178 PMC3782611

[16] Kaur K, Tandon H, Gupta AK, Kumar M. CrisprGE: a central hub of CRISPR/Cas-based genome editing. Database (Oxford). 2015;2015:bav055. Epub 2015/06/30. 10.1093/database/bav055 . 26120138 PMC4483309

[17] Bae S, Park J, Kim J-S. Cas-OFFinder: a fast and versatile algorithm that searches for potential off-target sites of Cas9 RNA-guided endonucleases. Bioinformatics (Oxford, England). 2014;30(10):1473–5. Epub 2014/01/24. 10.1093/bioinformatics/btu048 . 24463181 PMC4016707

[18] Park J, Bae S, Kim J-S. Cas-Designer: a web-based tool for choice of CRISPR-Cas9 target sites. Bioinformatics. 2015;31(24):4014–6. 10.1093/bioinformatics/btv537 26358729

[19] Naito Y, Hino K, Bono H, Ui-Tei K. CRISPRdirect: software for designing CRISPR/Cas guide RNA with reduced off-target sites. Bioinformatics. 2015;31(7):1120–3. Epub 2014/11/22. 10.1093/bioinformatics/btu743 . 25414360 PMC4382898

[20] Concordet JP, Haeussler M. CRISPOR: intuitive guide selection for CRISPR/Cas9 genome editing experiments and screens. Nucleic acids research. 2018;46(W1):W242–W5. Epub 2018/05/16. 10.1093/nar/gky354 . 29762716 PMC6030908

[21] Wang Y, Liu X, Ren C, Zhong G-Y, Yang L, Li S, et al Identification of genomic sites for CRISPR/Cas9-based genome editing in the Vitis vinifera genome. BMC plant biology. 2016;16(1):1–7. 10.1186/s12870-016-0787-3 27098585 PMC4839089

[22] Cheng J, Chen Y, Hu Y, Zhou Z, Hu F, Dong J, et al Fine mapping of restorer-of-fertility gene based on high-density genetic mapping and collinearity analysis in pepper (*Capsicum annuum* L.). Theoretical and Applied Genetics. 2020;133(3):889–902. Epub 2019/12/22. 10.1007/s00122-019-03513-y . 31863157

[23] Qin C, Yu C, Shen Y, Fang X, Chen L, Min J, et al Whole-genome sequencing of cultivated and wild peppers provides insights into *Capsicum* domestication and specialization. Proc Natl Acad Sci U S A. 2014;111(14):5135–40. 10.1073/pnas.1400975111 . 24591624 PMC3986200

[24] Kim S, Park M, Yeom SI, Kim YM, Lee JM, Lee HA, et al Genome sequence of the hot pepper provides insights into the evolution of pungency in Capsicum species. Nature genetics. 2014;46(3):270–8. 10.1038/ng.2877 . 24441736

[25] Kothari SL, Joshi A, Kachhwaha S, Ochoa-Alejo N. Chilli peppers—A review on tissue culture and transgenesis. Biotechnology Advances. 2010;28(1):35–48. 10.1016/j.biotechadv.2009.08.005 19744550

[26] Pozueta-Romero J, Houlne G, Canas L, Schantz R, Chamarro J. Enhanced regeneration of tomato and pepper seedling explants for Agrobacterium-mediated transformation. Plant Cell Tissue and Organ Culture. 2001;67(2):173–80.

[27] Rice P, Longden I, Bleasby A. EMBOSS: the European Molecular Biology Open Software Suite. Trends in genetics: TIG. 2000;16(6):276–7. Epub 2000/05/29. 10.1016/s0168-9525(00)02024-2 . 10827456

[28] Edgar RC. Search and clustering orders of magnitude faster than BLAST. Bioinformatics. 2010;26(19):2460–1. Epub 2010/08/17. 10.1093/bioinformatics/btq461 . 20709691

[29] Rizk G, Lavenier D. GASSST: global alignment short sequence search tool. Bioinformatics. 2010;26(20):2534–40. 10.1093/bioinformatics/btq485 20739310 PMC2951093

