# Supplementary figures and images for: Genome-wide identification and analysis of highly specific CRISPR/Cas9 editing sites in pepper (Capsicum annuum L.)
Source: PLoS One. 2020 Dec 29;15(12):e0244515. doi: 10.1371/journal.pone.0244515 (PMC7771699; doi:10.1371/journal.pone.0244515)

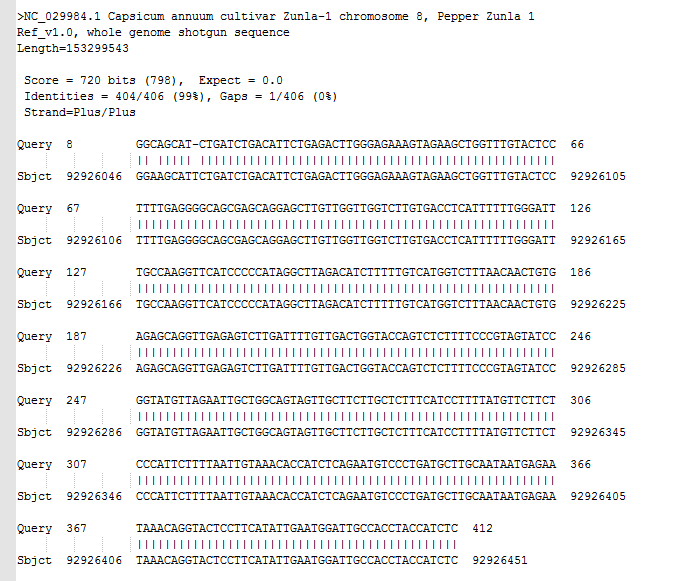


**protospacer**

**S1 Fig. Alignment of B7-1 sequence to the Zunla-1 reference genome.**

Supplement: S1 Fig — (DOCX) [file pone.0244515.s003.docx]
