# Supplementary material for: Genome-wide identification and analysis of highly specific CRISPR/Cas9 editing sites in pepper (Capsicum annuum L.)
Source: PLoS One. 2020 Dec 29;15(12):e0244515. doi: 10.1371/journal.pone.0244515 (PMC7771699; doi:10.1371/journal.pone.0244515)
Supplement: S1 Raw images — (PDF) [file pone.0244515.s004.pdf]

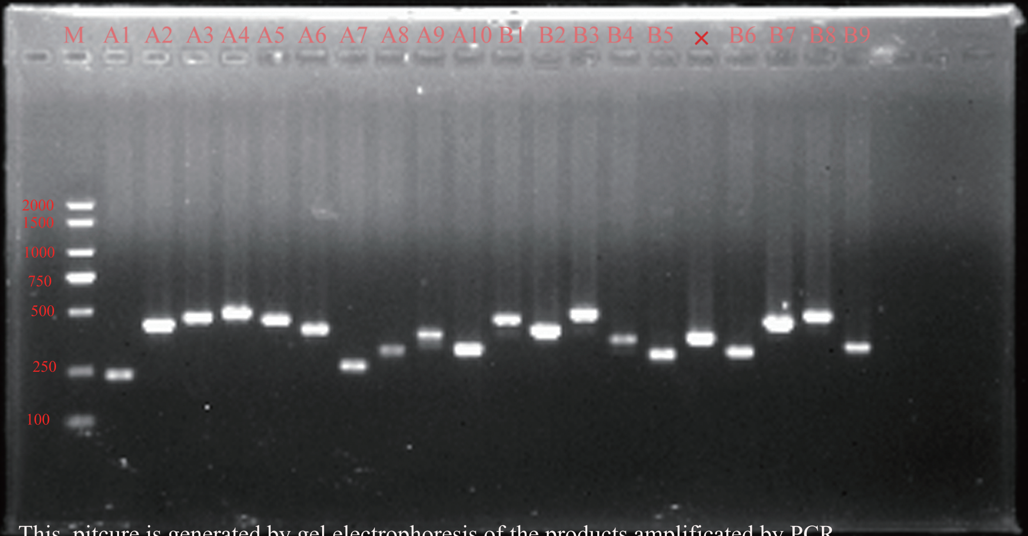

This pitcure is generated by gel electrophoresis of the products amplificated by PCR.
The M lane is marker DL2000 plus, and the sample from × lane is excluded from our final Figure 5.
